# Differentially expressed circulating miRNAs in postmenopausal osteoporosis: a meta-analysis

**DOI:** 10.1042/BSR20190667

**Published:** 2019-05-14

**Authors:** Elif Pala, Tuba Denkçeken

**Affiliations:** 1 Department of Medical Biology, Faculty of Medicine, SANKO University, Gaziantep, Turkey; 2 Department of Biophysics, Faculty of Medicine, SANKO University, Gaziantep, Turkey

**Keywords:** biomarker, circulating miRNAs, meta-analysis, Postmenopausal osteoporosis, Robust rank aggregation

## Abstract

MicroRNAs (miRNAs) have been proven to play a crucial role in postmenopausal osteoporosis (PMO), and studies on their diagnostic value have been increasing. In our study, we aim to identify the key miRNAs in the PMO that might be potential biomarkers. A comprehensive systematic literature search was conducted by searching PubMed, Web of Science, Embase and Cochrane Library databases. In the total of 16 independent miRNA expression studies which contained 327 PMO patients and 328 postmenopausal (PM) healthy control samples, miRNAs were evaluated by using robust rank aggregation (RRA) method. A statistically significant meta-signature of up-regulated hsa-miR-133a-3p (*P* = 1.38e−03) was determined. Then bioinformatics analysis to recruit putative target genes prediction of hsa-miR-133a-3p and pathway enrichment analysis to reveal what biological processes this miRNA may affect were conducted. It was indicated that pathways were commonly associated with adrenergic signaling in cardiomyocytes, adherens junction, PI3K-Akt signaling pathway and AMPK signaling pathway. Furthermore, STRING and Cytoscape tools were used to visualize the interactions between target genes of hsa-miR-133a-3p. Six genes were detected as hub genes among 576 targets which were CDC42, RHOA, EGFR, VAMP2, PIK3R2 and FN1. After Kyoto Encyclopedia of Genes and Genomes pathway analysis, it was detected that these hub genes were mostly enriched in signaling pathways and cancer. In this meta-analysis, it is stated that circulating hsa-miR-133a-3p may serve as a potential non-invasive biomarker and therapeutic target in PMO.

## Introduction

Osteoporosis is a systemic skeletal disorder, which is common in postmenopausal (PM) women, characterized by an increased risk of bone fragility and a decrease in bone mass [1]. Bone homeostasis requires a balance between bone-forming osteoblast cells and bone-resorbing osteoclast cells [2]. When this balance is impaired, normal bone remodeling cannot keep bone mass stable and leads to develop osteopenia and osteoporosis [3]. Traditionally, dual-X-ray absorptiometry (DEXA) which measures bone mineral density (BMD) is routinely used to assess the risk of fracture in osteoporosis [4].

MicroRNAs (miRNA) are short noncoding and single-stranded molecules in 18–24 nucleotides length which play key roles in translation and expression of genes via binding to the 3′untranslated region (3′UTR) of target messenger RNAs (mRNA) [5,6]. miRNAs have been identified as playing critical roles in biological processes (BPs) like differentiation and development; thus abundant studies associated with dysregulated miRNA expression in bone tissue and circulatory biofluids in osteoporosis [3]. According to these studies, miRNAs contribute to the pathogenesis of osteoporosis and therapeutic potential of them should be considered as they regulate several kinds of cells in bone homeostasis [3].

miRNA profiling datasets were arising rapidly with the improvement of high-throughput technologies. Using of different technological platforms and small sample size in studies results miRNA data sets show inconsistent results between various studies. In our study, we aimed to identify the key circulating miRNAs in the postmenopausal osteoporosis (PMO) that might be non-invasive potential biomarkers. Considering our aim and these problems, we found a substantial way to increase the statistical power of profiling data by combining the results of several studies. We combined these results via conducting a meta-analysis applying the robust rank aggregation (RRA) method [7], followed by pathway analysis, to identify miRNA dysregulation in PMO and the pathways that key miRNAs may affect [8]. The RRA strategy has been generated for comparison of different ranked gene lists and identification of commonly overlapping genes. This is a proper and efficient method for discrimination of statistically significant miRNA meta-signature and is particularly beneficial when experiments are performed by distinct technological platforms include different gene sets and full rankings of miRNAs are not available [9]. miRNA meta-signature investigation and identification of involved pathways would present potential targets for additional experimental studies of PMO development.

## Materials and methods

### Search strategies

We conducted a systematic literature search to identify miRNA expression profiling studies in PMO published up to 9 October 2018. The databases searched included PubMed, Web of Science, Embase and Cochrane Library through the MESH search headings ‘(miRNA OR microRNA OR miR) AND (osteoporosis)’. The searches were limited to English language studies and only full text published studies were included. We evaluated potentially relevant studies according to their titles and abstracts by using Rayyan which is a free application that dramatically speeds up the process of screening and selecting studies [10]. Moreover, each article was controlled to evaluate the publication type through its existed database manually.

### Study selection

The inclusion criteria for the selection of eligible studies were defined as: (1) circulating miRNA expression profiling on PMO patients; (2) have to compare PMO samples with PM healthy control samples; (3) miRNA expressions were reported to be profiled by using miRNA microarray, next-generation sequencing (NGS) or qRT-PCR; (4) have to report cut-off criteria of dysregulated miRNAs; (5) they reported fold changes (even if non-explicit); (6) have to report sample sizes. The exclusion criteria were defined as: (1) studies on tissues, cell lines and animals; (2) review literature and case reports.

Relevant papers were selected by two authors (E.P. and T. D.) and decided the list of studies be included carefully.

### Data extraction

From the full text and supplementary data of each study, the subsequent eligible information was collected and documented as first author, date of publication, country of study, sample sizes, tissue types, miRNA expression profiling assay type, number of probes, the list of dysregulated miRNAs and their related fold changes if given and cut-off criteria of dysregulated miRNAs. The authors directly contacted the correspondence of the studies to reach the gene lists which were not given in the full text and supplementary information. All miRNA names were standardized according to miRBase v22 by using *miRNAmeConverter* available in Bioconductor R package [11].

### Robust rank aggregation analysis

RRA method was used in our meta-analysis which is a free package in R software (http://cran.R-project.org/). This approach is utilized for comparison of different ranked gene lists, and is a definite and powerful approach for identification of differentially expressed miRNA integrated signature where a *P*-value would be assigned for each miRNA in the ranked lists to rerank these miRNAs and determine their significance. This method applies a probabilistic model for aggregation. The RRA method is robust to noise, and it facilitates the computation of significance probabilities to each of the elements in the final ranking [8,12]. All miRNAs from included studies were ranked with this method by using their *P*-values (*P*<0.05).

### Prediction of target gene differentially expressed miRNAs

In the present study MultiMiR package (http://multimir.ucdenver.edu/) was constructed to predict targets of miRNAs, which cover 14 databases. MultiMiR package was used to predict targets of miRNAs by DIANA-microT, ElMMo, MicroCosm, miRanda, miRDB, PicTar, PITA and TargetScan databases and also, validated targets of miRNAs were acquired from miRecords, miRTarBase and TarBase databases with the criterion of primary score listed in top 35 [13]. Only targets predicted by at least three algorithms or those validated by one database were kept for analyses [14].

### Functional enrichment analysis

The Database Annotation for Visualization and Integrated Discovery (DAVID) is an online program which allows functional annotation of the huge number of genes obtained from several genomic resources [15]. We used the DAVID database to perform gene ontology (GO) and Kyoto Encyclopedia of Genes and Genomes (KEGG) pathway analysis tools to implement the enrichment analysis. The consensus targets of miRNAs were used as input in screening GO and KEGG pathway analyses. *P*-values≤0.05 after Benjamini correction were considered enriched for the genes targeted by the selected miRNA [14].

### Protein–protein interaction network construction of the target genes

The STRING database (http://string-db.org/) is online software that aims to provide a crucial evaluation and integration of protein–protein interaction (PPI), including direct and indirect connections [16]. Cytoscape is one of the most favorite open-source software tools for the visual exploration of biomedical networks composed of protein, gene and other types of interactions [17]. The target genes were mapped to STRING with a confidence score>0.9 as a cut-off criterion to evaluate the PPI information and then interactions were visualized with Cytoscape. Node degree≥15 was set as the cut-off criterion to screen the hub genes. Furthermore, KEGG pathway analysis was applied to these hub genes by using DAVID with a *P*-values≤0.05 after Benjamini correction cut-off criterion.

## Results

### Literature search and included studies

**Figure 1:**
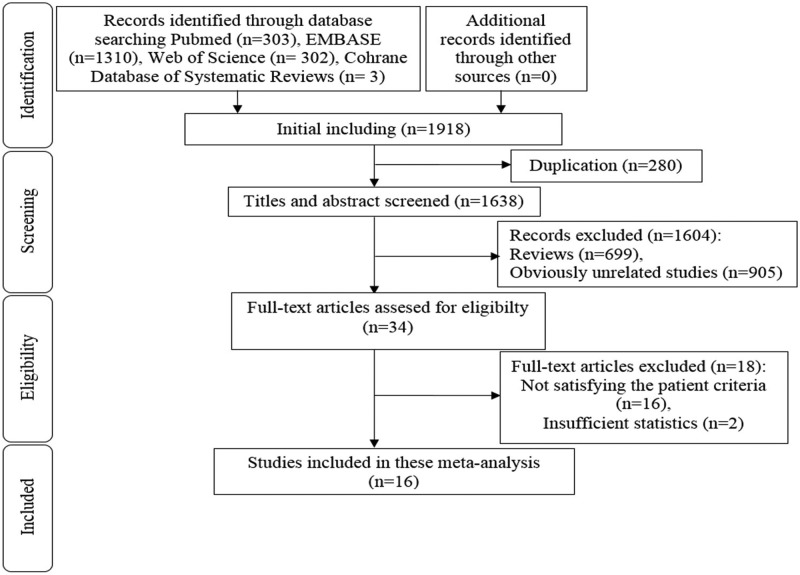
The flow diagram for study selection

The flow diagram demonstrating the strategy used to include in this meta-analysis is shown in Figure 1. 1918 possible relevant studies were detected in Pubmed, Web of Science, EMBASE and Cochrane databases considering our criteria. After eliminating duplicated publications, reviews and unrelated studies according to our inclusion/exclusion criteria, 34 articles met the eligibility. Finally following full-text analysis 16 articles included in this meta-analysis. The main information about the included studies was given in Table 1 [18–33].

**Table 1:** Characteristics of included miRNA profiling studies

No	Author	Date	Country	Sample type	# of samples (P/C)	Assay type	# of probes	Total	Up- regulate	Down- regulate	Cut-off criteria
**1**	Li Z	2018	China	Blood (serum)	20 (10/10)	qRT-PCR	1	1	1	0	*P*<0.05
**2**	Jiménez-Ortega RF	2017	Mexico	Blood (monocyte)	12 (6/6)	Affimetrix GeneChip miRNA 4.0 Array	2578	35	3	3	FC>0.5, *P*<0.05
**3**	Bedene A	2016	Slovenia	Blood (plasma)	74 (17/57)	qRT-PCR	9	1	1	0	*P*<0.05
**4**	Yavropoulou MP	2017	Greece	Blood (serum)	100 (70/30)	qRT-PCR	14	5	2	3	*P*<0.05
**5**	Meng J	2015	China	Blood	56 (32/24)	qRT-PCR	1	1	1	0	*P*<0.05
**6**	Li H	2014	China	Blood (serum)	80 (40/40)	qRT-PCR	3	2	1	1	*P*<0.01
**7**	Cao Z	2014	America	Blood (monocyte)	20 (10/10)	qRT-PCR	4	1	1	0	*P*<0.05
**8**	Wang Y	2012	America	Blood (monocyte)	20 (10/10)	TaqMan Human MicroRNA Array v1.0	365	2	2	0	*P*<0.05
**9**	Chen J	2016	China	Blood (serum)	29 (10/19)	qRT-PCR	15	4	0	4	*P*<0.05
**10**	Liu H	2017	China	Blood (plasma)	40 (20/20)	qRT-PCR	2	2	2	0	*P*<0.05
**11**	Chen H	2017	China	Blood (serum)	60 (30/30)	qRT-PCR	5	3	3	0	*P*<0.05
**12**	Chen C	2013	China	Blood	20 (10/10)	miRNA microarray, LC Sciences	721	7	3	4	*P*<0.05
**13**	Seeliger C	2014	Germany	Blood (serum)	60 (30/30)	qRT-PCR	13	9	9	0	*P*<0.05
**14**	Chen R	2018	China	Blood	18 (9/9)	qRT-PCR	150	14	4	7	*P*<0.05
**15**	Ramírez-Salazar EG	2018	China	Blood (serum)	40 (20/20)	TaqMan Array Human MicroRNA A+B Cards Set v3.0	754	7	7	0	FC≥2, *P*≤0.05
**16**	Jin D	2018	China	Blood	6 (3/3)	Sequencing (Illumina HiSeq platform)	NR	13	3	10	*P*<0.05

Abbreviations: C, Control; FC, fold change; P, patient.

### Differentially expressed miRNAs in postmenopausal osteoporosis vs. healthy postmenopausal controls

**Figure 2:**
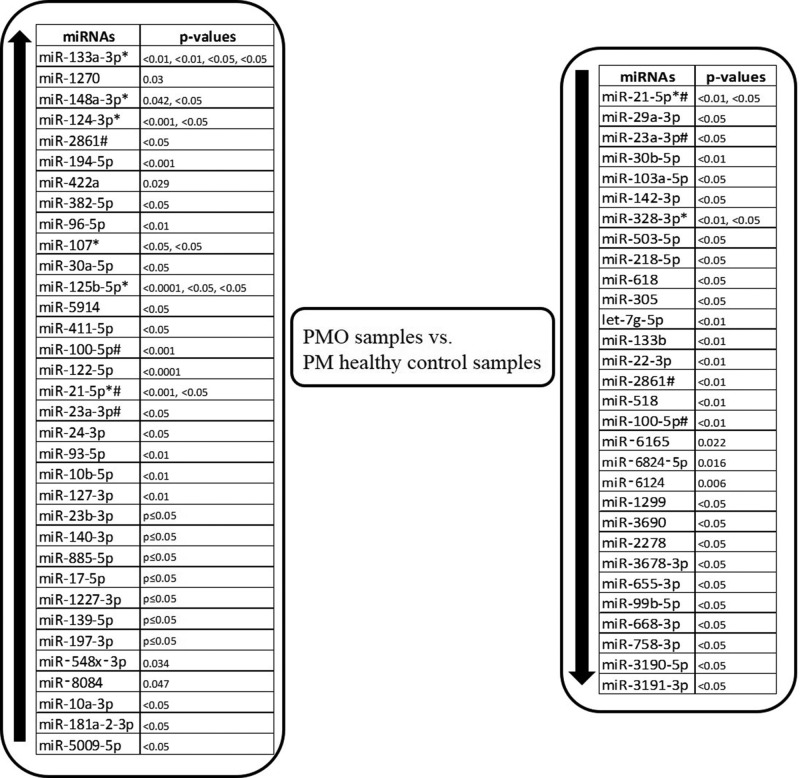
Up- and down-regulated miRNAs of PMO vs. PM healthy control samples in 16 included studies * indicates miRNAs reported in more than one study, # indicates miRNAs reported to be dysregulated in both directions.

In the included 16 miRNA expression profiling studies, 75 miRNAs were reported to be differentially expressed in groups of 327 PMO patients and 328 PM healthy controls. Among these miRNAs 32 were down-regulated, 43 were up-regulated and 10 miRNAs (hsa-miR-133a-3p, hsa- miR-148a-3p, hsa-miR-21-5p, hsa-miR-124-3p, hsa-miR-2861, hsa-miR-23a-3p, hsa-miR-328-3p, hsa-miR-107, hsa-miR-125b-5p and hsa-miR-100-5p) were reported in at least two studies. Furthermore four miRNAs (hsa-miR-21-5p, hsa-miR-2861, hsa-miR-23a-3p and hsa-miR-100-5p) were reported to be dysregulated in both directions (Figure 2).

Afterward, using the RRA method, we identified a statistically significant meta-signature of one up-regulated miRNA; hsa-miR-133a-3p considering the permutation *P*-value (1.38e−03). Only this miRNA was detected in two datasets of included studies by using RRA.

### Target prediction of differentially expressed hsa-miR-133a-3p

Target genes of hsa-miR-133a-3p were evaluated via bioinformatics analysis. The combination of eight different *in silico* predicted and three validated databases was systematically screened by using MultiMiR for miRNA–target interactions. According to our criterion, a total of 3153 target genes were predicted initially, and only 576 genes were essential for further studies.

### The enrichment analysis for predicted targets of hsa-miR-133a-3p

**Figure 3:**
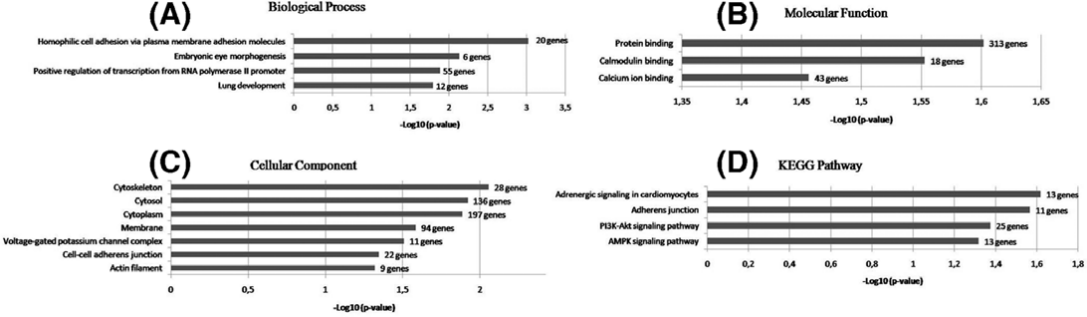
Enriched GO terms of 576 target genes obtained from the database for annotation (A) BP; (B) CC; (C) MF; (D) Enriched KEGG pathway

Using consensus target genes of hsa-miR-133a-3p enrichment analysis were performed to highlight the biological function of our PMO-miR meta-signature. Following the enrichment analysis by DAVID software, it was detected the most significantly enriched GO terms on BPs are homophilic cell adhesion via plasma membrane adhesion molecules, embryonic eye morphogenesis, positive regulation of transcription from RNA polymerase II promoter and lung development (Figure 3A). We listed all the BP, cellular components (CCs) (Figure 3B), molecular functions (MFs) (Figure 3C), and KEGG pathways (*P*<0.05 after Benjamini correction). Also, the KEGG pathway analysis showed that target genes were enriched in adrenergic signaling in cardiomyocytes, adherens junction, PI3K-Akt signaling pathway and AMPK signaling pathway (Figure 3D).

### PPI network and identification of hub genes

**Figure 4:**
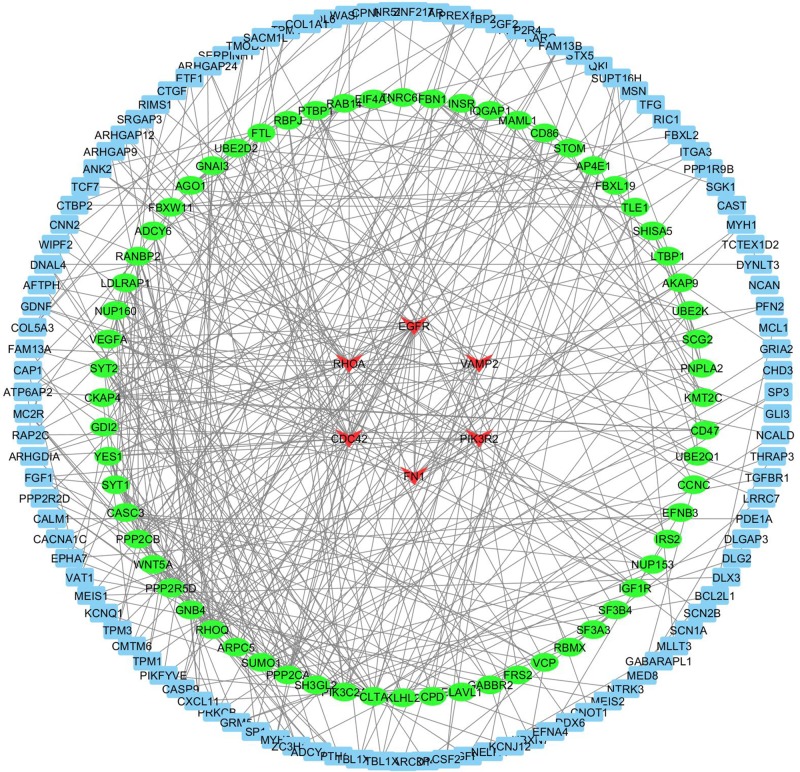
The PPI network of the target genes of miR–133a–3p from which six hub genes colored in red were identified according to the value of degrees

**Figure 5:**
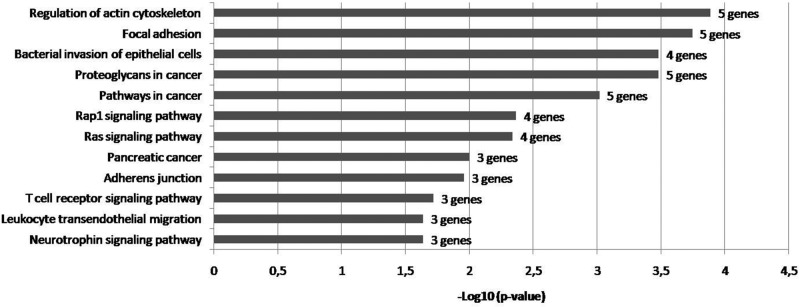
Enriched KEGG pathway of hub genes

Target genes were used to establish the PPI network by STRING, which composed of 572 nodes and 509 edges. Subsequently, we analyzed the STRING results using Cytoscape (Figure 4.) and six genes in the PPI network were identified as hub genes (node degree≥15). These included CDC42 (degree = 33), RHOA (degree = 25), EGFR (degree = 24), VAMP2 (degree = 19), PIK3R2 (degree = 16), and FN1 (degree = 15). KEGG pathway enrichment analysis of these hub genes was performed by DAVID. The pathway enrichment analysis revealed that hub genes were mostly enriched in signaling pathways and cancer (*P*-values≤0.05 after Benjamini correction) (Figure 5).

## Discussion

According to the World Health Organization, osteoporosis is one of the most common diseases, and 30–50% of all women in the world suffer fractures due to osteoporosis throughout their lives [34]. Circulating miRNAs are ideal non-invasive biomarkers as they are stable in body fluids and can easily be detected by using confirmed techniques for quantification [35]. miRNAs can regulate the expression of multiple mRNAs so that they are involved in almost all key BPs like proliferation, differentiation, migration and apoptosis [36]. There has been a growing interest in the regulation of miRNA studies for the development and progression of various diseases in recent years.

DEXA is a gold standard method for the measurement of BMD, used in the diagnosis of osteoporosis. To our knowledge, the present study is the first meta-analysis concerning PMO circulating miRNA expression profiles and identifying specific miRNA as a potential biomarker in PMO. Serum biomarker analyzing is beneficial as dysregulation of specific circulating miRNAs in the serum might present valuable potential diagnostic predictors for PMO.

Although there are some studies in the literature on PMO and circulating miRNAs, no meta-analysis is available, and consistent and precise data cannot be reached. In the present study, we aimed to use meta-analysis to recognize consistently dysregulated miRNAs, which were mentioned in profiling results to be candidate biomarkers for PMO. To overcome inconsistent results of miRNA dysregulation in PMO patients reported in the literature, RRA method was performed in the present study. Ranked list of miRNAs from each included study was aggregated into a single gene ranking and analyzed using the RRA method, which approved hsa-miR-133a-3p as the only statistically significant miRNA associated with PMO (*P* = 0.00138).

Dysregulation of miR-133a-3p has been identified in several types of cancers, such as bladder [37], colorectal [38], osteosarcoma [39], non-small cell lung [40] and esophageal cancers [41]. Two genes encode human mature miR-133a: MIR133A1 for miR-133a1 at 18q11.2 and MIR133A2 for miR-133a2 at 20q13.33. These two genes encode different premature miRNAs but produce the identical mature miR-133a sequence. Genetic researches also discovered the association of 18q11.2 to osteoporosis-related traits [42] and the linkage of 20q13 to bone phenotypes [43,44]. Wang et al. aimed to identify important miRNAs in human circulating monocytes of 20 high and low BMD PM women and found significant up-regulation of miR-133a in the low BMD group by both array and qRT-PCR analyses. They suggest that miR-133a in circulating monocytes is a potential biomarker for PMO [25]. Besides, Li et al. searched the levels of miR-133a in the plasma of 120 Chinese PM women who were divided into three groups of normal, osteopenia and osteoporosis according to the T-scores. Up-regulation of miR-133a was validated in the plasma of osteoporosis and osteopenia cases versus the normal group and suggested a potential use of miR-133a as sensitive and plasma biomarker for PMO [18].

Also, there are studies available concerning circulating miRNAs in PMO based on different inclusion patient criteria in the literature. Studies on the circulating miRNAs in PMO patients that were compared with the PM women without osteoporosis were included in this meta-analysis. As noted in Figure 1, studies comparing PMO patients with osteoarthritic controls [45] and PMO women with and without fracture (control) [46,47] were excluded as they did not meet patient criteria.

MiR-133a was initially examined as a muscle-specific miRNA involved in the regulation of muscle-cell differentiation and pathogenesis of heart disease [48]. Afterward, it was determined that miR-133a plays a crucial role in bone morphogenesis and fracture healing [49]. RUNX2 is involved in the differentiation of mesenchymal stem cells into osteoblast and bone formation [50,51]. MiR-133a can inhibit the secretion and expression of bone formation-promoting substances and other molecules related to osteoblast differentiation via binding to RUNX2 [52]. It was shown that the expression level of miR-133a was significantly increased and RUNX2 protein expression level was significantly decreased in bone tissues of patients with fracture nonunion, suggesting that the miR-133a expression level was closely related to fracture healing [53].

In an included study to our meta-analysis, *in vitro* experiments demonstrated that miR-133a was up-regulated during osteoclastogenesis and overexpression of this miRNA promoted RANKL induced differentiation of RAW264.7 and THP-1 cells into osteoclasts. And also *in vivo* experiments carried out in the present study showed that osteoclastogenesis related factors levels changed in serum, lumbar spine BMD increased and bone histomorphology changed in miR-133a knockdown ovariectomized rats. These results indicated that miRNA-133a is involved in the regulation of PMO by promoting osteoclast differentiation [18].

Target gene prediction for miR-133a-3p was performed by using MultiMiR. 576 genes were detected as the target of this miRNA and KEGG pathway analysis has shown that these genes were mostly enriched in adrenergic signaling in cardiomyocytes and adherens junction. Some other reports in the literature that show the association of these two pathways with osteoporosis [54,55]. Furthermore, target genes of miR-133a-3p were used to establish the PPI network by using Cytoscape and six genes in the PPI network were identified as hub genes (degree≥15). These included CDC42, RHOA, EGFR, VAMP2, PIK3R2 and FN1. Increased activity of osteoclasts is related with several bone diseases, including PMO. After adherence to the bone, the osteoclasts become polarized and reorganize their cytoskeleton and membrane to form unique domains including the sealing zone. This zone is a dense ring of F-actin-rich podosomes delimiting the ruffled border, where protons and proteases are secreted to demineralize and degrade the bone matrix, respectively. These processes are dependent on the activity of small GTPases. CDC42 and RHOA are members of Rho GTPases that regulate podosome assembly and their organization into the sealing zone [56]. Epidermal growth factor receptor (EGFR) is one member of the transmembrane growth factor receptor proteins [57]. It has been shown that the EGFR network was closely associated with bone biology and pathology; for example, it affected osteoblastic bone formation and bone mass in different ways. Recently, Zhu et al. found that the EGFR system suppressed osteoblast differentiation and have crucial functions in skeletal homeostasis [58]. In compliance with all this information, the KEGG pathway analysis of the hub genes showed that these genes are mostly enriched in regulation of actin cytoskeleton and focal adhesion.

## Conclusions

In conclusion, hsa-miR-133a-3p seems to be a potential biomarker for PMO, since its levels are up-regulated in patients suffering from PMO. This miRNA would enable the analysis of osteoporosis without requiring DEXA so that radiation exposure will be reduced in these patients. Thus, a deeper examining of the role of miRNAs in osteoporosis can inspire critical implications for the early diagnosis and prevention of osteoporosis. It can also provide unique opportunities to develop novel pharmacologic approaches for osteoporosis.

## Abbreviations

BMD: bone mineral density
BP: biological process
CC: cellular component
DAVID: Database Annotation for Visualization and Integrated Discovery
DEXA: dual-X-ray absorptiometry
EGFR: epidermal growth factor receptor
GO: gene ontology
KEGG: Kyoto Encyclopedia of Genes and Genomes
MF: molecular function
miRNA: microRNA
mRNA: messenger RNA
PM: postmenopausal
PMO: postmenopausal osteoporosis
PPI: protein–protein interaction
RRA: robust rank aggregation
